# Supplementary figures and images for: Child abuse associates with increased recruitment of perineuronal nets in the ventromedial prefrontal cortex: a possible implication of oligodendrocyte progenitor cells
Source: Mol Psychiatry. 2021 Nov 19;27(3):1552–61. doi: 10.1038/s41380-021-01372-y (PMC9095471; doi:10.1038/s41380-021-01372-y)

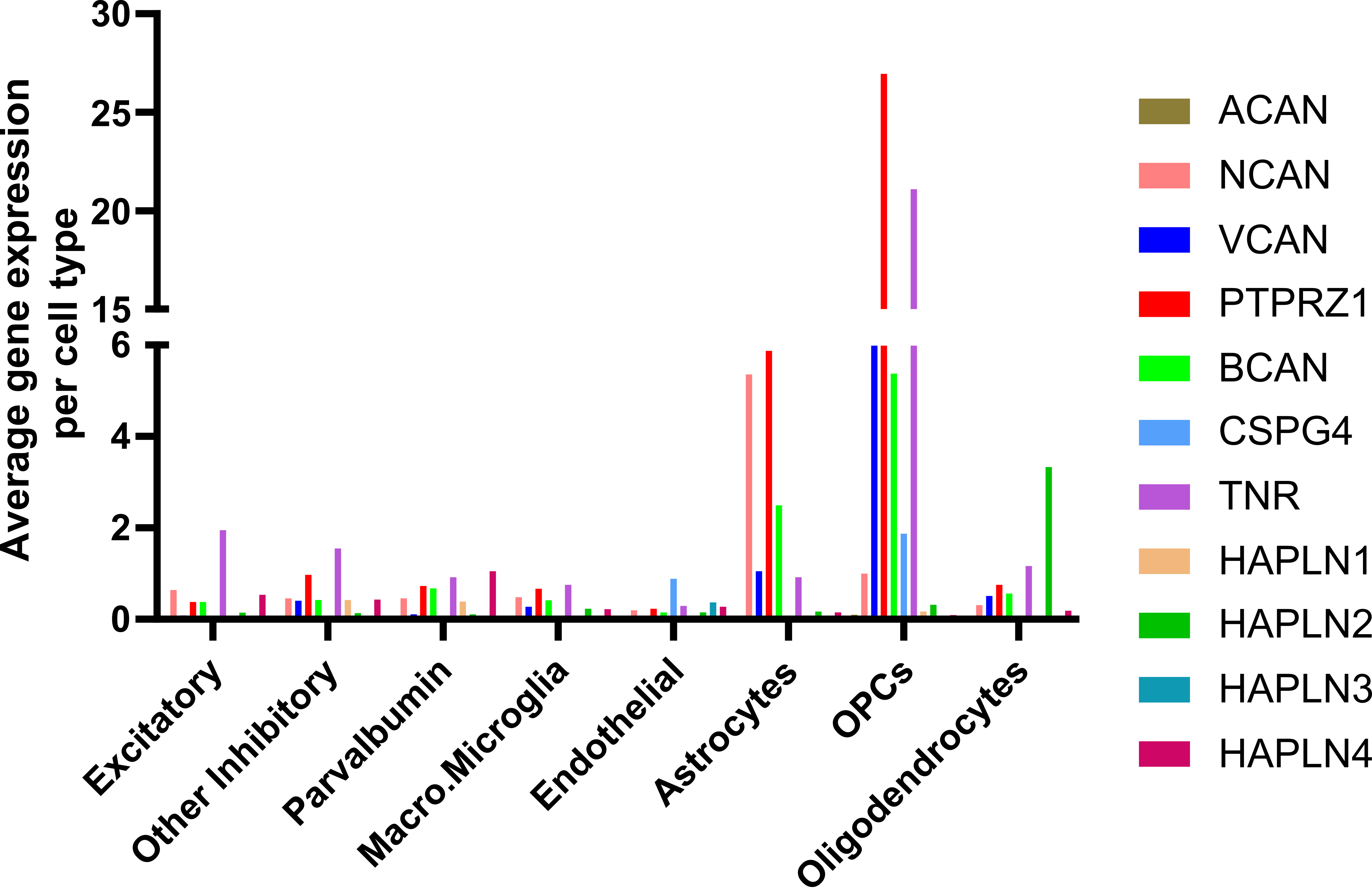

Supplement: Supplementary file 1 — Supplementary Figure 1 [file 41380_2021_1372_MOESM1_ESM.png]
