# Supplementary material for: Child abuse associates with increased recruitment of perineuronal nets in the ventromedial prefrontal cortex: a possible implication of oligodendrocyte progenitor cells
Source: Mol Psychiatry. 2021 Nov 19;27(3):1552–61. doi: 10.1038/s41380-021-01372-y (PMC9095471; doi:10.1038/s41380-021-01372-y)
Supplement: Supplementary file 3 — Supplementary information [file 41380_2021_1372_MOESM3_ESM.docx]

**Supplementary Figure legends**

**Supplementary Figure 1**. Average normalized expression of PNN-related genes (See Figure 2) in major cell types of the human prefrontal cortex. Data derives from a previous snRNA-seq study published by our group^20^ (see Supplementary Tables 30-31 of this study). A strong enrichment of some of these genes is observed in OPCs, in particular VCAN (versican), PTPRZ1 (phosphacan) and TNR (tenascin-R).
